# The relationship between life regrets and well-being: a systematic review

**DOI:** 10.3389/fpsyg.2024.1515373

**Published:** 2024-12-19

**Authors:** Jocelyn A. Rutledge, Jadyn D. Williams, Meaghan A. Barlow

**Affiliations:** Department of Psychology, Wilfrid Laurier University, Waterloo, ON, Canada

**Keywords:** life regret, well-being, aging, coping, health

## Abstract

**Introduction:**

The aim of the current study is to examine the association between life regret and well-being, through a systematic review.

**Methods:**

Four different databases (PsycINFO, Web of Science, ProQuest, Dissertations & Theses Global, and ERIC) were used to source 31 relevant articles, published between 1989 and 2018.

**Results:**

We conclude that experiencing greater life regret is associated with negative effects on various aspects of well-being, such as life satisfaction and depressive symptoms. Although the impact of life regret on well-being is suggested to vary across individual differences in lived experience, age- and gender-related findings remain mixed across studies. This inconsistency may be partly due to the varying protective factors and coping mechanisms individuals use, which may mediate the relationship between life regrets and well-being. Protective factors include the degree of engagement or disengagement towards regret reversal, social comparison, appraisal, and interpretation.

**Discussion:**

However, these conclusions are not definite, as the measurement of regret and well-being are inconsistent across studies and there is limited diversity in study samples. Moreover, further research including diverse populations and more standardized measures is necessary to strengthen existing links and identify mediators that could serve as modifiable protective factors between life regret and well-being.

**Systematic Review Registration:**

https://osf.io/hy7xj.

## Introduction

Many of us will, at some point in our lives, be confronted with the question, “What is your biggest regret?.” This question often prompts deep reflection on our past decisions and experiences that have shaped our present lives, for better or for worse. Although the concept of living life without regrets is often idealized, given the many choices and divergent paths we must navigate, it is typically unattainable to live a regret-free life. This is evidenced by past research, demonstrating that 90% of individuals typically experience severe life regrets (Wrosch et al., 2005). Such regrets can feel uncomfortable, often signalling perceived flaws in personal choices or a lack of control (Roese and Summerville, 2005). Thus, it seems apparent that dwelling on foregone opportunities could have significant impacts on a person’s well-being—but what does the extant research reveal about these impacts? To answer this question, the present systematic review aims to summarize and analyze the existing body of literature examining the association between life regrets and well-being.

### Conceptualization of life regrets

Regret is a complex emotional experience that arises from the engagement of higher-order cognitive processes, including reflection, evaluation, and imagination (Landman, 1987). Regret is closely tied to counterfactual thinking, a process of mentally reconstructing past events and contemplating “what if” possibilities, enabling us to evaluate how different actions or circumstances might have led to alternative outcomes (Epstude and Roese, 2008). This self-focused, upward thinking process often elicits negative emotional responses, conceptualized as regrets (Roese et al., 2007; Zeelenberg and Pieters, 2007). Specific regret-related emotions have been identified and categorized into despair (e.g., helpless, desperate, and sad), hot (e.g., angry, embarrassed, and ashamed), and wistful emotions (e.g., contemplative, nostalgic, and sentimental; Gilovich et al., 1998). Consequently, regret has further been described as a ‘counterfactual emotion’ (Kahneman and Miller, 1986).

This review focuses on life regrets–a particularly enduring type of regret that arises from significant, pivotal life events.^1^ Life regrets are a psychological phenomenon encompassing both cognitive and affective components (Landman, 1987; Wrosch and Heckhausen, 2002), and are often characterized as one’s most severe, major, important, burdensome, or existential regret in the context of life circumstances (Bauer and Wrosch, 2011; Farquhar et al., 2013; Metha et al., 1989; Reker and Woo, 2011; Schmidt and Van der Linden, 2013; Stewart and Vandewater, 1999; Tassone et al., 2019). Life regrets arise through reflective processes, where individuals assess their past decisions and compare their present life circumstances to imagined outcomes, had those choices been different (Roese, 1997). This counterfactual thinking process can trigger negative thoughts and emotions, arising from discrepancies between an unsatisfying present reality and idealized alternative states.

Life regret experiences often reflect normative life priorities and personal values, such as interpersonal relationships, professional endeavors, and personal growth (Roese and Summerville, 2005). From the perspective of goal attainment, life regrets arise when individuals reflect on their inability to achieve certain goals, wishing they had made different choices that might have led to the successful attainment of these goals (Jokisaari, 2003; Lecci et al., 1994; Wrosch and Heckhausen, 2002). Lifespan theories propose that developmental goals persist throughout one’s life across normative time points (Heckhausen et al., 2010). The internalization of these normative and societal expectations establishes a standard and framework through which individuals evaluate their success in life. An individual’s success, or lack thereof, in accomplishing these developmental tasks has thus been suggested to influence what people identify as their most significant life regret (Jokisaari, 2004; Wrosch and Heckhausen, 2002). Accordingly, major developmental areas, like education, career, and family are often the most cited domains of people’s most severe life regrets (Gilovich and Medvec, 1994, 1995; Jokisaari, 2004; Lecci et al., 1994; Roese and Summerville, 2005).

This developmental perspective of life regret is further evidenced by age-related patterns in life regret experiences. Specifically, the content of people’s life regrets tends to reflect their current developmental stage of life, including presently salient life domains and former developmental goals (Jokisaari, 2004; Kinnier and Metha, 1989). For example, younger adults typically report more leisure and relationship regrets, while older adults tend to focus on regrets related to work and family (Jokisaari, 2003, 2004). Further, older age, specifically around the age of retirement, has been recognized as a phase where significant life review commonly occurs (Butler, 1963). In this vein, older adults also begin to recognize they have fewer instrumental resources to undo and resolve their life regrets, due to normative, physical, and temporal constraints (Lecci et al., 1994; Wrosch and Heckhausen, 2002). Thus, life regrets may be particularly salient and impactful for older adults and in later life.

Importantly, normative expectations of lifespan development differ with respect to various other contextual factors, including gender and sociocultural background (Baltes, 1987; Sagiv and Schwartz, 2022). Societally imposed gender roles and cultural norms further influence the perceived expectations, opportunities, and constraints that impact an individual’s ability to attain their goals (Hagestad, 1990; Wrosch et al., 2003). In this vein, differences in the content and degree of life regrets have also been shown to reflect evolving trends of normative gender expectations, suggesting the importance of cohort and historical context in shaping people’s regret experiences (Newton et al., 2012).

### Life regrets and well-being

Given the pervasive and enduring nature of life regrets, it is important to understand the impact of these experiences on our well-being. The nature of life regret experiences might be indicative of how these events impact us psychologically, emotionally, and physically. Counterfactual thoughts are recognized as an underlying mechanism in the experience of regret (Kahneman and Miller, 1986) and have been associated with greater psychological and emotional distress (Branscombe et al., 2003; Callander et al., 2007; Gilbar and Hevroni, 2007; Landman et al., 1995). In this vein, viewing life regret as a reflection of unattained goals may underlie negative impacts on well-being (Messersmith and Schulenberg, 2010; Lecci et al., 1994; Jokisaari, 2003; Jokisaari, 2004). Life regret has been significantly associated with internal attribution (Zeelenberg et al., 1998), and the feeling of personal responsibility that accompanies intense regret has been shown to evoke negative self-related emotions (Mandel, 2003). Feeling burdened by self-blame, particularly regarding life regrets related to major developmental domains, can hinder people from being fully satisfied with the trajectory of their current lives.

Despite the psychological and emotional consequences of feeling burdened by life regret, various coping mechanisms may be protective of negative cognitive and emotional experiences. For example, psychological mechanisms that function to promote more positive and adaptive interpretations and appraisals of life regret might be more effective in reducing the intensity of negative emotional responses (Kahneman and Miller, 1986). Further, from the theoretical perspective of goal attainment, the therapeutic efficacy of actively engaging in efforts to undo a regret may be contingent upon the possibility of reversal (Beike et al., 2009; Wrosch and Heckhausen, 2002). Overall, a combination of contextual factors, including individual differences, might influence how people can effectively manage thoughts and feelings associated with their life regrets, resulting in diverse effects on overall well-being.

In summary, as with any experience unique to humans, the impact of life regrets on well-being varies widely, influenced by personal differences and life experiences, and shaped by how we perceive and address past choices.

### The present review

Given the high prevalence and commonplace nature of life regret across individuals and throughout the lifespan, it is important to understand the impact of these experiences on well-being. The purpose of the present systematic review is to identify links between the experience of life regret and well-being. We seek to explore how the experience of life regret impacts various facets of well-being, and to identify the specific dimensions of life regret that contribute to these effects. As the following review will demonstrate, research in this area is relatively limited and marked by considerable variability in the conceptualization and measurement of life regret across studies. Therefore, this review aims to highlight the diverse methodological approaches used in the study of life regret, to describe how life regret affects well-being across the diversities in the human experience—such as age, gender, and culture—, and identify strategies that can mitigate these effects. Finally, this review concludes by highlighting gaps in the existing research and recommended areas for future inquiries.

## Method

In conducting this systematic review, we followed the Preferred Reporting Items for Systematic Reviews and Meta-Analyses (PRISMA) guidelines (Moher et al., 2009; Page et al., 2021). We chose to conduct a systematic review rather than a meta-analysis for two reasons: (1) our aim was to describe the full body of empirical research examining the association between life regrets and well-being, and (2) the aforementioned body of research is remarkably heterogeneous in terms of measurement, making meaningful quantitative aggregation both difficult and ill-advised. In the following sections, we describe our efforts to optimize transparency and openness, search strategy, inclusion and exclusion criteria, screening and data extraction procedures, and finally, our data analysis plan.

### Transparency and openness

We used several strategies to adhere to the Transparency and Openness Promotion Guidelines (Nosek et al., 2015). First, following the search stage, this systematic review was pre-registered using the Generalized Systematic Review Registration Form (Van den Akker et al., 2023) on the Open Science Framework (OSF; https://osf.io/hy7xj; Barlow et al., 2024). Further, as previously noted, this review was conducted in accordance with PRISMA guidelines suggested for conducting a systematic review (Moher et al., 2009; Page et al., 2021). Finally, as noted throughout the methods section, where possible, research materials have been made available on OSF (https://osf.io/4va7j/; Barlow et al., 2024).

### Search strategy

The search strategy was based on an informal literature review to obtain common terms within the literature exploring life regret. As this area is limited, the authors agreed upon doing the most expansive search of the term ‘regret’ possible to locate all relevant work. Accordingly, the database search for the term “regret*” in any field available in the database (e.g., title, abstract, keywords, full text, etc.) was conducted in July 2023 using ProQuest Database to search the following interfaces: APA PsychINFO; Web of Science; ProQuest Dissertations & Theses Global; ERIC. No limiters were used in this search. This review will also include grey literature searchable in the identified databases (e.g., theses). As recommended by Page et al. (2021), we validated our search strategy by identifying a set of articles that fully met the inclusion criteria and confirmed they were all in the initial search results.

A total of 19,572 records were identified in the database search. These records were imported into HubMeta software (a web-based data entry system for meta-analysis; https://hubmeta.com/). Once imported, HubMeta identified 10,562 duplicates, which were removed. Thus, a total of 9,010 records were screened for inclusion.

### Inclusion and exclusion criteria

All identified records were reviewed to determine if they met the following criteria:

1. Life regret measure: Studies had to include a measure of regret specified explicitly as a life regret, long-term regret, severe regret, or greatest regret. Studies examining short-term/decisional regret or anticipated regret were excluded.
2. Well-being measure: Studies had to include a measure of well-being (e.g., life satisfaction, depressive symptoms, quality of life, physical well-being).
3. Participants: Studies with adult subjects (18 years or older) were included. Studies specifically recruiting children or adolescents were excluded.
4. Type of research: Empirical and quantitative research was included. Qualitative research, including qualitative dissertations, systematic reviews, and theoretical papers, were excluded.
5. Language: Articles were included only if they were written in English, as this is the language spoken by all three authors.^2^

### Screening

**Figure 1:**
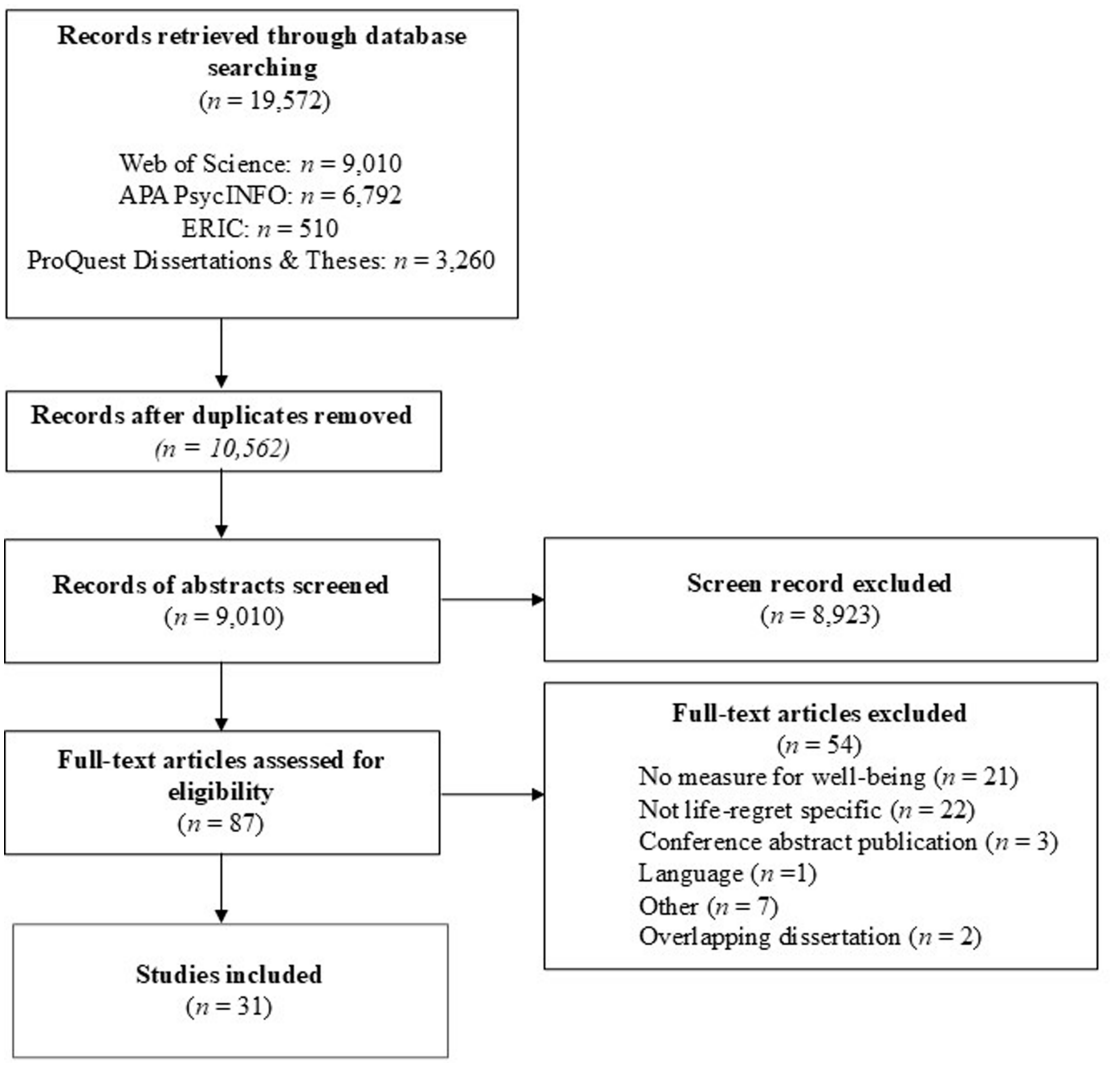
PRISMA diagram for the systematic review detailing the database searches and screening process.

As previously mentioned, a total of 19,572 records were identified. Following the removal of duplicates, 9,010 records were screened for inclusion. To adhere to scientific rigor, the screening process was conducted by two independent screeners (the co-first authors: JDW & JAR). Discrepant and borderline decisions were discussed and agreed upon between screeners. Screening was conducted in two steps. First, title screening was conducted in which each article title and abstract were examined, confirming the study adhered to the inclusion criteria. Only basic bibliography fields (i.e., title, abstract, authors, journal, year) were visible during title and abstract screening. This step excluded 8,923 records. Second, the screeners read the full text of the remaining 87 records to confirm the inclusion criteria was met for each study. This step excluded 56 records, resulting in a final dataset consisting of 31 articles (see Figure 1 for PRISMA diagram). Further, a file indexing all the excluded articles has been made available on OSF (https://osf.io/4va7j/; Barlow et al., 2024).

### Data extraction and synthesis

Data extraction was done in parallel by the two first co-authors (JDW & JAR) to ensure reliability of the data, while also considering feasibility. Disagreements were reconciled through discussions including all authors (Cohen’s Kappa = 0.93). The extracted data has been made available on OSF (https://osf.io/4va7j/; Barlow et al., 2024). For each included article, the following information was extracted:

1. Citation Information: Authors, publication year, title, journal, publication type (peer-reviewed, book chapter, other/specify).
2. Sample Information: Population (e.g., older adults, students, retired individuals, etc.), sample description, sample size, design (e.g., cross-sectional, longitudinal, experimental, other/specify), sample characteristics (age, ethnicity, race, gender, sex, education, socioeconomic status, marital status), and location of study.
3. Methods: Research questions, hypotheses, methods/procedures, regret constructs measured, regret scales used, well-being constructs measured, well-being scales used, other constructs measured, and other scales used.
4. Results: Findings summary, non-significant findings, themes (i.e., aging, coping, cross-cultural effects, gender effects, general trends, protective factors, risk factors, other).
5. Introduction: Theory/rationale of the study.
6. Discussion: Impacts, limitations, future directions.
7. Study Quality: The study quality assessment was adapted from the Appraisal tool for Cross-Sectional Studies (AXIS; Downes et al., 2016) and the Strengthening the Reporting of Observational Studies in Epidemiology (STROBE) Checklist for cross-sectional studies (Von Elm et al., 2007). The study quality inventory has been made available on OSF (https://osf.io/4va7j/; Barlow et al., 2024).
8. Additional notes (optional).

As we did not have specific a-priori questions, but rather a general aim to describe the available research examining the association between life regrets and well-being, we implemented a bottom-up approach. Accordingly, the decision of which themes to include was based on (1) the number of articles in each theme, (2) the total number of themes, and (3) the importance of the conclusions from each theme.

The study findings and methodological information were synthesized within and across the various themes to identify patterns. The data synthesis was conducted collaboratively by the two first co-authors (JDW & JAR). All data and codebooks will be made available on OSF (https://osf.io/4va7j/; Barlow et al., 2024).

## Results

Overall, the existing literature indicates life regrets are associated with poorer well-being across 31 studies. These associations are detailed in Table 1. In the following sections, we will summarize the findings across the following themes: sample characteristics, and methodological characteristics, regret measures, well-being measures, protective factors, age-related findings, gender-related findings.

**Table 1:** Summary of measures and results pertaining to regret and well-being.

Citation	Regret measure(s)	Well-being measure(s)	General results: regret and well-being
Bauer and Wrosch (2011)	Perceived opportunities to undo life regret Social comparison direction	Positive affect Negative affect Cold symptoms	Downward regret-related social comparisons with low perceived opportunities to undo life regret = ↑ Positive affect Upward regret-related social comparison with low perceived opportunities to undo life regret = Lowest positive affect Downward regret-related social comparisons with low perceived opportunities to undo life regret = ↑ Positive affect, ↓ Cold symptoms over time Change in positive affect mediated the combined effect of social comparison direction and opportunities on change in cold symptoms over time. Effects independent of age.
Choi and Jun (2009)	Content Intensity (Frequency × Degree) Causal attribution	Depressive symptoms Current life stressors Health status Serious daily life problems	↑ Marriage regret intensity = ↑ Loneliness ↑ Loss/grief regret intensity = ↑ Depressive symptoms ↑ Behavioral/self regret intensity = ↑ Depressive symptoms, ↑ Loneliness ↑ Health status regret intensity = ↑ ADL/IADL impairments
DeGenova (1993)	Frequency	Life satisfaction	↑ Regretfulness = ↓ Life satisfaction
Dijkstra and Barelds (2008)	Content Interpretation	Life satisfaction Positive/negative affect Self-esteem Physical well-being	Presence of life regret = ↓ Physical well-being, ↓ Positive affect, ↑ Negative affect, ↓ Self-esteem, ↓ Life satisfaction Reported fully coming to terms with regret = Highest life satisfaction, most positive affect, least negative affect, highest self-esteem and best physical health Reported having not come to terms with regret = Lowest life satisfaction, least positive affect, most negative affect, lowest self-esteem and worst physical health Put the best face on things = Intermediate well-being
Farquhar (2008); Farquhar et al. (2013)	Content Engagement in undoing Opportunity to undo	Everyday activities Emotional well-being Retirement satisfaction	Favorable opportunities + high engagement in undoing regrets = ↑ Retirement satisfaction (at baseline), ↑ Everyday activities (at baseline and over time) Favorable opportunities + low engagement in undoing regrets = ↓ Everyday activities, ↓ Retirement satisfaction Unfavorable opportunities + high engagement in undoing regrets = ↓ Retirement satisfaction over time
Funderburk (2008)	Presence Content	Life satisfaction Depression	Presence of life regret = ↓ Life satisfaction ↑ Depression = Increased chances for regret
Herriot et al. (2018)	Intensity	Physical health problems Normative diurnal cortisol secretion	↑ Regret intensity = ↓ Self-compassion ↑ Regret intensity = ↑ Cortisol secretion (moderated by low self-compassion)
Isenberg (2007)	Intensity Frequency Number of regrets	Health Happiness Depression	↑ Regret intensity and frequency = ↑ Depression, ↓ Happiness, ↓ Health status Regret intensity and frequency did not predict depression after controlling for personality and dispositional variables.
Jokisaari (2003)	Content Evaluation Timeline	Life satisfaction Physical symptoms Depressive symptoms Negative affectivity	↑ Regret consequences = ↓ Life satisfaction, ↑ Physical symptoms, ↑ Depressive symptoms ↑ Regret importance-disappointment = ↓ Life satisfaction, ↑ Physical symptoms, ↑ Depressive symptoms, ↑ Negative affect ↑ Regret impact on life = ↓ Life satisfaction
Jokisaari (2004)	Content	Life satisfaction Depressive symptoms	↑ Education and work-related regrets = ↓ Life satisfaction ↑ Self-related regrets = ↑ Depressive symptoms
Kinnier and Metha (1989)	Content	Life Satisfaction	↑ Risk-related regret = ↓ Life satisfaction
Kourakis (2008)	Amount	Death Anxiety Depressive symptoms Health status	↑ Regret = ↑ Depressive symptoms, ↓ Health status, ↑ Death anxiety
Lecci et al. (1994)	Content Evaluation	Life satisfaction Depression Negative affectivity	↑ Number of regrets = ↑ Depression ↑ Regret investment + disappointment = ↑ Depression, ↓ Life satisfaction ↑ Progression with regret + positive outcome expectancy = ↑ Life satisfaction ↑ Time + energy investment in regret = ↓ Life satisfaction ↑ Investment + ↑ disappointment = ↑ Depression
Lee and Ryu (2018)	Content Intensity (Frequency × Degree)	Depression Current life stressors Health status Serious daily life problems	Leisure and addiction regrets (Americans) = ↑ Geriatric depression Health and career regrets (Koreans) = ↑ Geriatric depression
Lewis and Borders (1995)	Content Degree	Life satisfaction Health	↑ Regret degree = ↓ Life satisfaction, ↓ Health
Mckee et al. (2005)	Presence	Psychological health status Physical health status Social well-being	Having life regrets = ↓ Psychological health, ↓ Positive affect, ↓ Social well-being
Metha et al. (1989)	Presence Content	Life satisfaction	Education regrets and not taking risk regrets = ↓ Life satisfaction
Neimeyer et al. (2011)	Degree	Psychological well-being Self-esteem Fear of death	↑ Life regret = ↓ Self-esteem, ↓ Psychological well-being, ↑ Fear of death
Newall et al. (2009)	Content Number of regrets Frequency	Health conditions Life satisfaction Past depression	↑ Regret frequency = ↑ Health conditions, ↓ Life satisfaction, ↑ Past depression ↑ # of regrets = ↑ Health conditions, ↓ Life satisfaction, ↑ Past depression ↑ # of regrets = ↓ Interpretive control
Newton et al. (2012)	Regret Type Number of regret types	Psychological well-being	↑ # of regret types = ↓ Life satisfaction
Pethtel (2012); Pethel and Chen (2014)	Content Intensity	Life satisfaction Positive/negative affect Emotion Regulation	↑ Regret intensity = ↓ Life satisfaction, ↓ Positive affect, ↑ Negative affect, ↓ Emotional stability, ↑ Emotional suppression
Reker and Woo (2011)	Intensity	Physical health status Life satisfaction Depression Fear of aging	↑ Regret intensity = ↓ Life satisfaction, Depression, ↑ Fear of aging
Schmidt and Van der Linden (2013)	Regret induction Habitual levels of counterfactual thoughts	Sleep onset latency Total wake time in bed Total sleep time	Regret induction in participants with habitually high levels of regret = Delayed sleep onset
Seiden (2001)	Content Impact	Quality of life Life satisfaction	↑ Personal action regret = ↓ Quality of personal life, ↑ Long-term distress ↑ Family and romantic regrets = ↓ Family satisfaction, ↓ Personal well-being
Stewart and Vandewater (1999)	Content Regret-related life change	Psychological distress Physical well-being Life satisfaction	Did not make regret-related change = ↑ Depressed, ↑ Anxious, ↓ Physical health Rumination and effective instrumentality mediated the association between regret and well-being for those who did not make regret-related life changes
Tassone et al. (2019)	Content Temporal distance	Emotional response to regret	↑ Age = ↓ Negative regret-related emotion Older adults = Higher levels of positive than negative emotions when reflecting on regrets Younger adults = Similar levels of positive and negative regret-related emotion Emotional stability mediated the relationship between age and regret-related negative affect
Tibbett and Ferrari (2018)	Content Intensity	Life satisfaction	↑ Regret across all domains = ↓ Life satisfaction Life regret mediated the association between indecision and lower life satisfaction
Torges et al. (2005)	Regret Type Current interpretation	Life satisfaction Physical well-being Recent positive mood	↑ Regret types = ↓ Life satisfaction, ↓ Physical health, ↓ Recent positive mood Has not come to terms with regret = ↓ Life satisfaction, ↓ Recent positive mood Have come to terms with regret < Put the best face on things < Not come to terms with regret
Wrosch et al. (2007)	Content Intensity Writing intervention	Diurnal cortisol rhythms Acute physical symptoms Negative affect Cold symptoms Sleep problems	↑ Regret intensity = ↑ Cortisol dysregulation, ↑ Acute physical health problems, Volume and steeper morning increase in cortisol secretion, ↑ Cold symptoms Regret intensity at baseline = ↑ Cold symptoms over time Adaptive social–cognitive writing task intervention = ↓ Regret intensity
Wrosch et al. (2005)	Content Intensity Intrusive thoughts about regrets Perceived opportunities to undo	Depressive symptoms Physical health problems Negative affect Life satisfaction	↑ Intrusive thoughts about life regrets = ↑ Negative affect, ↑ Depressive symptoms, ↑ Health problems, ↓ Life satisfaction ↑ Disengagement (only in older adults) = Regret intensity, ↓ Depression, ↓ Health problems Depressive symptoms mediated the association between regret intensity and physical health in older adults
Wrosch and Heckhausen (2002)	Content Intensity Regret-induced emotions	Self-esteem Life satisfaction	↑ Regret intensity = ↑ Despair-related emotions ↑ Regret-related despair emotions = ↓ Self-esteem

### Sample characteristics

For an overview of the sample characteristics of the included papers, please refer to Table 2. In general, the samples predominantly consisted of White individuals with approximately 50% of the studies were conducted in the United States. About half of the studies reported no information about the sample’s race or ethnicity. Among those that did, most participants were Caucasian, with fewer individuals identifying as African American or Black, Hispanic or Latino, Asian, and Native American. One study focused specifically on individuals of Korean descent, living in South Korea. Regarding gender, about half of the studies reported equal representation of men and women, while the other half predominantly included samples where self-identifying females accounted for over 70% of the participants. Additionally, five studies exclusively sampled women, while one focused solely on men. One-third of the studies reported the participants’ education levels, with most samples consisting of individuals holding bachelor’s degrees. Marital status was reported in nearly one-third of the studies, with approximately half of the participants being currently married. Moreover, two studies focused exclusively on either all married or all single individuals. Of the included studies, 53.33% recruited older adults samples (*n* = 16; i.e., samples including only older adults), 20% recruited age-comparative samples (*n* = 6; i.e., samples including multiple age groups), 16.67% recruited adult lifespan samples (*n* = 5; i.e., samples including a continuous age range spanning the majority of the adult lifespan), and 13.33% recruited other types of samples (*n* = 4, i.e., undergraduates samples, middle-aged adults). Lifespan samples varied across studies, collecting participants as young as 16 years old up to 99 years old. Twenty-six studies had sample sizes ranging from approximately 100 to 300, while the remaining 4 studies utilized sample sizes exceeding 600. Very few studies included a power analysis or justification for their sample size. Some studies specifically sampled male veterans, hospice patients, and individuals working in professional careers.

**Table 2:** Sample characteristics.

First author, Year	Place of publication	Sample Description	Design	Age (years)	Ethnicity/Race (% White Caucasian)	Gender (% women) or Sex (% female)	Education (% holding bachelor’s degrees or # years)	Married (%)	Country
Bauer and Wrosch (2011)	Personality and Social Psychology Bulletin	Study 1: 104 younger and older adults Study 2: 51 older adults	Longitudinal	Study 1: Young: 18–35 (M = 25.25, SD = 4.83) Old: 60+ (M = 67.85, SD = 6.39) Study 2: 60–87 (M = 71.73, SD = 7.95)	N/A	Study 1: 61% female Study 2: 61% female	Study 1: 50% Study 2: 55%	Study 1: 39% in an intimate relationship Study 2: 53% in an intimate relationship	Canada
Choi and Jun (2009)	Aging & Mental Health	213 older, low-income adults	Cross-sectional	58–95 (M = 74.5, SD = 8.81)	27.7%	75.1% women	18.8%	26.3%	USA
DeGenova (1993)	Educational Gerontology	122 retirees	Cross-sectional	54–91 (M = 72.1)	N/A	66% female	12.5 years	68%	USA
Dijkstra and Barelds (2008)	Journal of Research in Personality	3,579 Dutch women, (lifespan sample)	Cross-sectional	16-81(M = 45.58, SD = 10.64)	N/A	100% female	N/A	78%	Netherlands
Farquhar (2008); Farquhar et al. (2013)	International Journal of Aging & Human Development	289 retirees	Longitudinal	44–77 (M = 58.94, SD = 4.94)	N/A	55% female	15.08 years	N/A	Canada
Funderburk (2008)	University of California	1,256 elders	Cross-sectional	75–98 (M = 81, SD = 4)	92%	54% female	13.82 years	46%	USA
Herriot et al. (2018)	Journal of Behavioral Medicine	233 older adults	Cross-sectional	59–93 (M = 75.57, SD = 7.75)	N/A	60.94%	25.75%	50.21%	Canada
Isenberg (2007)	Concordia University	Phase 1: 111 older adults Phase 2: Subsample of 71 from phase one	Cross-sectional	65–92 (M = 74.14, SD = 6.12)	N/A	60% women	14.13 years	49.5%	Canada
Jokisaari (2003)	Journal of Research in Personality	176 adults (lifespan sample)	Cross-sectional	19–82 (M = 42.6; SD = 19.5)	N/A	52% women	N/A	N/A	Finland
Jokisaari (2004)	Journal of Adult Development	176 adults (lifespan sample)	Cross-sectional	19–82 (M = 42.6; SD = 19.5)	N/A	52% women	N/A	N/A	Finland
Kinnier and Metha (1989)	Counselling and Values	316 adults (3 cohorts)	Cross-sectional	Cohort 1: 20–29 Cohort 2: 35–55 Cohort 3: 64+	93%	55% women	41%	57%	USA
Kourakis (2008)	Florida State University	19 male veterans	Cross-sectional	45–84 (M = 62.05, SD = 8.57)	52.6%	0% female	27.4%	42.1%	USA
Lecci et al. (1994)	Journal of Personality and Social Psychology	155 community college students	Cross-sectional	18–59 (M = 28.5, SD = 8.3)	N/A	72% women	N/A	N/A	USA
Lee and Ryu (2018)	The International Journal of Aging and Human Development	234 low-income older adults (USA: 130; Korean: 104)	Cross-sectional	65–95 (USA: M = 72.71, SD = 8.87; Korean: M = 74.61, SD = 7.13)	USA: 73.1%	USA: 74.6% female Korean: 92.3% female	USA: 33.1% Korean: 2.9%	USA: 35.4% Korean:48.1%	USA, Korea
Lewis and Borders (1995)	Journal of Counseling and Development	152 single women in professional careers	Cross-sectional	35–60 (M = 43.74)	86.8%	100% women	95.4%	0%	USA
McKee et al. (2005)	British Journal of Clinical Psychology	152 older adults living in nursing homes	Cross-sectional	64–98 (M = 84.2, SD = 7.10)	N/A	78.2% female	N/A	12.7%	UK
Metha et al. (1989)	Psychology of Women Quarterly	178 women (lifespan sample)	Cross-sectional	Young: 20–29 Middle: 35–55 Old: 65+ years	92%	100% women	N/A	51%	USA
Neimeyer et al. (2011)	Death Studies	153 hospice patients	Cross-sectional	39–99 (M = 73.8, SD = 12.4)	65%	54% women	N/A	52%	USA
Newall et al. (2009)	International Journal of Aging & Human Development	228 older adults	Cross-sectional	79–98 (M = 84.99, SD = 4.29)	N/A	62% women	10.47 years	38%	Canada
Newton et al. (2012)	Sex Roles	313 female graduates (3 cohorts)	Cross-sectional	Cohort 1(1951/52): 65–70 (M = 66) Cohort 2 (1972): 44–50 (M = 46) Cohort 2 (1992): 23–30 (M = 26)	87%	100% female	100%	Cohort 1: 98% Cohort 2: 90% Cohort 3: 28%	USA
Pethtel (2012)	Bowling Green State University	119 middle to older aged adults	Cross-sectional	39–76 (M = 52.94, SD = 7.80)	79.8%	71.4% female	38.6%	73.9%	USA
Reker and Woo (2011)	SAGE Open	120 older adults	Cross-sectional	52–93 (M = 73.6)	N/A	52% women	33%	62%	Canada
Schmidt and Van der Linden (2013)	Cognitive therapy and research	176 undergraduate psychology students	Experimental	17–45 (M = 20.94, SD = 4.18)	N/A	88% women	N/A	N/A	Switzerland
Seiden (2001)	ProQuest Dissertations Publishing	Study 1: 725 graduates Study 2: 561 males from Study 1 Study 3: 1512 graduates	Cross-sectional	No range reported Study 1: M = 39, SD = 5.4 Study 2: N/A Study 3:M = 37, SD = 9.6	Study 1: 92% Study 2: N/A Study 3: 82%	Study 1: 23% female Study 2: 0% female Study 3: 30.5% female	Study 1, 2, 3: 100%	Study 1, 2, 3: 100%	USA
Stewart and Vandewater (1999)	Journal of Personality and Social Psychology	Study 1: 83 middle-aged women Study 2: 76 from Study 1	Longitudinal	Study 1: 36 (at baseline) Study 2: 37 (at baseline)	N/A	Study 1 and 2: 100% women	Study 1 and 2: 100%	Study 1: 86% Study 2: 74%	USA
Tassone et al. (2019)	Psychology and Aging	620 adults (lifespan sample)	Cross-sectional	18–92 (M = 50.7)	84%	55% female	41%	N/A	USA
Tibbett and Ferrari (2018)	North American Journal of Psychology	2,271 adults	Cross-sectional	No range reported M = 42.26, SD = 13.32	80.8%	60% female	76.9%	46.7%	USA
Torges et al. (2005)	Journal of Research in Personality	259 older adults	Cross-sectional	60–65 (M = 61)	93%	48.6% women	0%	78%	USA
Wrosch et al. (2007)	Psychology and Aging	Study 1: 183 older adults Study 2: 103 older adults	Study 1: Cross-sectional; Study 2: Experimental	Study 1: 63–94 (M = 72.31, SD = 5.91) Study 2: 60–87 (M = 71.77, SD = 7.50)	N/A	Study 1: 52% female Study 2: 65% female	Study 1 = 32% Study 2 = 51%	N/A	Canada
Wrosch et al. (2005)	Psychology and Aging	Study 1: 120 younger and older adults Study 2: 150 younger, middle-aged, and older adults	Cross-sectional	Study 1: Young: 19–35 (M = 22.47, SD = 2.91) Old: 55–89 (M = 69.67, SD = 7.78) Study 2: M = 50.06, SD = 20.05) Young: 18–85 Middle: 35–59 Old: 60–85)	N/A	Study 1: 56% female Study 2: 53% male	Study 1: 46% Study 2: 51%	N/A	Canada
Wrosch and Heckhausen (2002)	Psychology and Aging;	122 young, middle-aged, and older adults	Cross-sectional	20–87 (M = 49.48, SD = 18.30)	N/A	55% female	N/A	N/A	Germany

### Methodological characteristics

The present review includes 26 (86.7%) cross-sectional studies, three (10%) longitudinal studies and two studies (6.7%) involving an experimental design. The average study quality was 17.94 (*SD* = 1.21); for more information, see OSF: https://osf.io/4va7j/. The majority of the cross-sectional studies utilized self-report questionnaires, with only five employing semi-structured or structured interviews. The included longitudinal studies investigated the impact of various coping mechanisms over 4 months [i.e., social comparison (Bauer and Wrosch, 2011)], engagement over 3 years (Farquhar et al., 2013), and regret-related life changes over 11 years (Stewart and Vandewater, 1999) on changes in affectivity, psychological well-being, and physical health indicators over time. Regarding experimental designs, one study involved a regret induction (Schmidt and Van der Linden, 2013) and the other examined the impact of a writing intervention on the relationship between life regret and well-being (Wrosch et al., 2007). Both experimental studies examined physical well-being measures as outcome variables.

### Regret measures

There are significant differences in how life regrets are conceptualized and measured across the reviewed literature. The variability in the operationalization of this concept is substantial, with minimal consistency across studies.

Nearly all studies collected information on the content of participants’ life regrets, though the methods used to collect this information varied across studies. Most studies asked participants to reflect on their lives and report their life regret experiences, often using descriptors such as major, greatest, most long-term, severe, or burdensome regret (Farquhar et al., 2013; Isenberg, 2007; Lee and Ryu, 2018; Newall et al., 2009; Pethtel, 2012; Seiden, 2001; Schmidt and Van der Linden, 2013; Tassone et al., 2019; Wrosch and Heckhausen, 2002; Wrosch et al., 2005, 2007). Other studies framed the concept of life regrets in alternative ways, for example, characterizing them as unfulfilled goals (Jokisaari, 2003, 2004; Lecci et al., 1994), opportunities not pursued (Newton et al., 2012; Torges et al., 2005), alternative lifestyle patterns (Metha et al., 1989; Newton et al., 2012; Torges et al., 2005), things one wishes they had handled differently (Choi and Jun, 2009; Dijkstra and Barelds, 2008; Farquhar et al., 2013; Stewart and Vandewater, 1999), or things they would have done to improve their life today (Funderburk, 2008). In some items, life regret was described to participants, and in others, a definition of regret was not provided, allowing participants to form their own interpretations of the concept (Isenberg, 2007).

Life regret items also varied in their use of open or closed-ended response methods. The majority of studies prompted participants to provide open-ended responses, either through face-to-face interviews including verbal recounts of life regret experiences (Choi and Jun, 2009; Isenberg, 2007; Newall et al., 2009; Lee and Ryu, 2018; Mckee et al., 2005), or through self-report surveys, allowing participants to describe one to three of their most significant life regrets (Herriot et al., 2018; Bauer and Wrosch, 2011; Dijkstra and Barelds, 2008; Farquhar et al., 2013; Funderburk, 2008; Jokisaari, 2003, 2004; Lecci et al., 1994; Newton et al., 2012; Schmidt and Van der Linden, 2013; Seiden, 2001; Stewart and Vandewater, 1999; Tassone et al., 2019; Torges et al., 2005; Wrosch et al., 2005, 2007; Wrosch and Heckhausen, 2002). Subsequently coding open-ended responses into regret categories that reflect fundamental life domains is a widely used practice in the literature. The following domains were most commonly used to categorize life regrets: education, occupation/work, family, romance, leisure, friendship, health, finance and personal/self. These domains are repeatedly cited as representing the most common life regret experiences (Dijkstra and Barelds, 2008; Lecci et al., 1994; Roese and Summerville, 2005; Schmidt and Van der Linden, 2013). When regret content was measured in a closed-ended format, participants were typically provided with a series of statements, each pertaining to a specific regret, and were asked to rate or indicate feelings of regret for each domain (DeGenova, 1993; Funderburk, 2008; Isenberg, 2007; Kourakis, 2008; Lewis and Borders, 1995; Metha et al., 1989; Pethtel, 2012; Tibbett and Ferrari, 2018). These categories similarly reflected the most common life domains eliciting regret, as described above. One simplified method of collecting regret was asking participants if they experienced life regret in a binary yes/no response format (McKee et al., 2005).

Another categorization approach involved coding regret responses into domains specific to the aims of the study. For example, Stewart and Vandewater (1999) categorized life regrets into whether they related to pursuing traditional versus non-traditional gender roles for women. Alternatively, some studies compared personal to work-related life regrets (Newton et al., 2012; Seiden, 2001), while others explored life regret in existential contexts, such as death and end-of-life review (Neimeyer et al., 2011; Reker and Woo, 2011). Torges et al. (2005) differentiated between regrets about lifestyle changes from regrets relating to missed opportunities. Separating regret responses into omission versus commission regrets also emerged as a common categorization across studies (Jokisaari, 2003; Seiden, 2001; Wrosch et al., 2005, 2007).

Although most studies used original life regret items, previously developed life regret scales are periodically referenced. Scales included the: Life Review Index (DeGenova, 1993), R questionnaire (Tomer and Eliason, 2005), 12-item Life Domain Regret Inventory (Roese and Summerville, 2005), Regrets Regarding Life Circumstances Scale (Metha et al., 1989), and Existential Regret Scale (Reker and Parker, 1999). Furthermore, after participants were asked to report their life regret, most studies also integrated Likert-type items to measure specific dimensions of life regret experiences. These dimensions include the intensity and frequency of life regret experiences, emotions elicited by life regrets, and an individual’s interpretation and appraisal of their life regrets.

Five studies examined regret frequency (Choi and Jun, 2009; DeGenova, 1993; Lee and Ryu, 2018; Newall et al., 2009; Wrosch et al., 2005), assessing how often one has regretful thoughts or experiences feelings of regret. Numerous studies also measured the temporal distance since the life regret event occurred (Farquhar, 2008; Farquhar et al., 2013; Jokisaari, 2003; Tassone et al., 2019; Wrosch et al., 2007).

Several studies included measures of regret intensity or degree, in which participants were asked to rate the strength or extent of regretful feelings for each specific life regret (Choi and Jun, 2009; Isenberg, 2007; Lee and Ryu, 2018; Lewis and Borders, 1995; Pethtel, 2012; Wrosch and Heckhausen, 2002; Wrosch et al., 2007). In one study, qualitative responses taken from interviews were coded by researchers to derive regret intensity ratings (Isenberg, 2007). Validated measures of regret intensity included the Life Regret Scale (Pethtel, 2012) and the Existential Regret Scale (Reker and Parker, 1999; Reker and Woo, 2011). Intrusive thoughts about life regrets were additionally used as a measure of regret intensity (Wrosch et al., 2007), as well as the number of life regrets or regret types (Kourakis, 2008; Lecci et al., 1994; Newall et al., 2009; Newton et al., 2012).

Self-report measures of regret-induced emotions were also used as indicators of regret intensity. The most commonly used regret-related emotions included the categories of despair-related, hot, and wistful emotions (Gilovich et al., 1998). Participants were asked to report how strongly they experience each emotion when thinking about their life regrets (Tassone et al., 2019; Wrosch and Heckhausen, 2002; Wrosch et al., 2005; Wrosch et al., 2007). Most approaches focused specifically on negative affectivity (Wrosch and Heckhausen, 2002; Wrosch et al., 2005; Wrosch et al., 2007), however, Tassone et al. (2019) examined both positive and negative emotions.

In light of regret appraisal and interpretation, several studies measured the opportunity to undo life regrets; that is, the likelihood that the negative consequences of the provided life regret can and will be undone (Bauer and Wrosch, 2011; Farquhar et al., 2013; Jokisaari, 2003; Wrosch et al., 2005). Some studies measured regret disengagement, specifically, the amount of effort one invests in undoing the regret (Farquhar et al., 2013; Wrosch et al., 2005), as well as perceived changeableness and control one has over their life regrets (Farquhar et al., 2013; Jokisaari, 2003; Lecci et al., 1994; Torges et al., 2005; Wrosch and Heckhausen, 2002). Two studies explored acceptance, categorizing participants as either not coming to terms with their regret, putting the best face on things, or fully coming to terms with and accepting the outcome of the life regret (Dijkstra and Barelds, 2008; Torges et al., 2005). Other life regret dimensions included causal attributions (e.g., societal barriers, family, personal responsibility; Choi and Jun, 2009), ratings of regret impacts (e.g., distress, disappointment; Jokisaari, 2003; Lecci et al., 1994), and ratings of one’s and others’ perceived importance (Jokisaari, 2003; Lecci et al., 1994). Social comparison was assessed by asking participants to evaluate how their regret compared to that of their aged peers (Bauer and Wrosch, 2011; Seiden, 2001).

### Well-being measures

#### General psychological well-being

Throughout the literature, various measures were used to examine psychological well-being. General measures of an individual’s psychological health status were used in two studies (Neimeyer et al., 2011; Mckee et al., 2005), and included the psychological domain of the World Health Organization’s Quality of Life Scale (WHOQOL Group, 1998), the Philadelphia Geriatric Centre Morale Scale (Lawton, 1975), and the General Health Questionnaire (Goldberg and Williams, 1988). However, throughout the literature, more specific measures of psychological well-being are predominantly used. Across studies, the primary measures of psychological well-being included life satisfaction, depression, anxiety, and affect.

#### Life satisfaction

Eighteen studies assessed life satisfaction. The most commonly used scale to measure life satisfaction was Diener et al.’s (1985) Satisfaction with Life Scale (*n* = 10; Dijkstra and Barelds, 2008; Jokisaari, 2003, 2004; Lecci et al., 1994; Lewis and Borders, 1995; Newton et al., 2012; Pethtel, 2012; Tibbett and Ferrari, 2018; Torges et al., 2005, Wrosch et al., 2005). Three other studies used items from the Life Satisfaction Index-A (LSI-A; DeGenova, 1993; Newall et al., 2009) and Index-Z (Reker and Woo, 2011), both developed by Neugarten et al. (1961). Four studies measured life satisfaction with a single item (Funderburk, 2008; Metha et al., 1989; Stewart and Vandewater, 1999; Wrosch and Heckhausen, 2002), while others assessed satisfaction with regard to more specific domains of life. For example, Farquhar et al. (2013) assessed retirement satisfaction at two time points: immediately after retirement and then at a three-year follow-up. Moreover, Seiden (2001) assessed personal and work-life satisfaction.

#### Depression and anxiety

Twelve studies included measures of depression or depressive symptoms. More specifically, studies most commonly employed Radloff’s (1977) Center for Epidemiological Studies Depression Scale (*n* = 4; Isenberg, 2007; Lecci et al., 1994; Newall et al., 2009; Wrosch et al., 2005), Beck et al. (1961) Depression Inventory (*n* = 2; Jokisaari, 2003, 2004), Zung’s (1965) Self-Rating Depression Scale (*n* = 2; Reker and Woo, 2011; Stewart and Vandewater, 1999), and Sheikh and Yesavage’s (1986) Geriatric Depression Scale (*n* = 2; Choi and Jun, 2009; Lee and Ryu, 2018). In addition, smaller measures of depression involved categorical responses regarding recent feelings of depression and the frequency of specific depressive symptoms (n = 2 Funderburk, 2008; Kourakis, 2008).

Two studies examined anxiety as an outcome variable. Stewart and Vandewater (1999) utilized the Zung’s (1971) Self-Rating Anxiety Scale. Kourakis (2008) used the revised version of the Collett-Lester Fear of Death & Dying Scale (Lester, 1994) to assess death anxiety (i.e., fear of death and dying of oneself and others).

#### Affect

Ten studies included scales measuring affect, particularly using positive and negative affect as primary indicators of subjective well-being. The Positive and Negative Affect Schedule (PANAS), originally created by Watson et al. (1988), was frequently utilized (Bauer and Wrosch, 2011; Farquhar, 2008; Farquhar et al., 2013; Pethtel, 2012; Torges et al., 2005; Wrosch et al., 2007), along with MacKinnon et al.’ (1999) shortened version (Dijkstra and Barelds, 2008). Similar in approach, McKee et al. (2005) implemented Lawton’s (1994) Apparent Affect Rating Scale (AARS). Another approach to assessing affect involved the Memorial University of Newfoundland’s Scale of Happiness (MUNSH; Kozma and Stones, 1983), which evaluates the balance between one’s positive and negative affect (Isenberg, 2007). An alternative measure collecting only negative affect, employed by Lecci et al. (1994), included the use of the NEO Five Factor Inventory (NEO-FFI; Costa and McCrae, 1989) measuring levels of neuroticism, which was interpreted as negative affectivity-emotionality. Also aimed at specifically measuring negative affect, but not previously piloted, Jokisaari (2003) asked participants to rate the extent to which they currently felt nervous, anxious, or unhappy.

#### Physical health

Studies commonly assessed health status by either providing a list of various health conditions for participants to choose from (Choi and Jun, 2009; Dijkstra and Barelds, 2008; Jokisaari, 2003) or by asking participants to rate their general state of health through a single-item question (Isenberg, 2007; Kourakis, 2008; Mckee et al., 2005; Reker and Woo, 2011). Similar in format, Lewis and Borders (1995) utilized two questions from Baruch et al. (1983), each regarding one’s overall health and energy levels. Wrosch et al. (2005) and Herriot et al. (2018) employed a checklist adapted from a prior study on Midlife in the United States (MIDUS; Wrosch et al., 2000) which includes questions concerning seven common health issues and their treatments, expected to be influenced by distress and experienced across age. In contrast, experiencing acute physical or cold symptoms in the recent weeks was also used as an indicator of participants current health status (Bauer and Wrosch, 2011; Wrosch et al., 2007). Unique measures of health aimed towards older participants include impairments in activities of daily living (ADL: Katz et al., 1963; IADL: Lawton and Brody, 1969; Cho et al., 2011; Herriot et al., 2018; Lee and Ryu, 2018), the Barthel scale for dependency (Mahoney and Barthel, 1965; Mckee et al., 2005), and measures of activity level from the Everyday Activities Questionnaire (Pushkar et al., 1997; Farquhar et al., 2013). In addition, the General Attitudes Toward Aging scale (Reker and Woo, 2011) assesses fears associated with aging, reflecting concerns individuals may currently experience or fear to experience in the future. Furthermore, sleep quality served as an indicator of one’s physical well-being and was assessed by the Insomnia Severity Index (Blais et al., 1997; Schmidt and Van der Linden, 2013) or the Sleep Quality Index (Buysse et al., 1989; Wrosch et al., 2007). Additional indicators of physical well-being included measures of daily cortisol levels at different time points (Herriot et al., 2018; Wrosch et al., 2007).

#### Other well-being measures

Two studies also measured self-esteem (Neimeyer et al., 2011; Wrosch and Heckhausen, 2002), captured by the Self-Esteem Scale of Rosenberg’s (1965). Other measures of psychological well-being included intrusive thoughts about personal problems (Wrosch and Heckhausen, 2002), social well-being (McKee et al., 2005), and effective social functioning Index of Adult Adjustment (Picano, 1989; Schmidt and Van der Linden, 2013).

In summary, there is much variation in the facets and scales of well-being used across studies. Despite the negative impacts that life regrets are observed to have on well-being (Table 1), other variables seem to mediate this link and are further described as protective factors.

### Protective factors

The literature points to various factors that differentiate individuals whose life regrets lead to adverse effects on their well-being from those who exhibit psychological resilience despite these experiences. We have organized this literature into four categories: (1) goal engagement and disengagement, (2) interpretation and appraisal, (3) social comparisons, and (4) individual differences.

#### Goal engagement and disengagement

Several studies suggest life regrets can be akin to failed goal attainment, implying that a life regret may reflect feelings similar to those associated with a significant unmet goal. In general, the research suggests that individuals can protect their well-being from life regret through actively changing the circumstance that led to the regret and/or disengaging both behaviorally and mentally from the regret experience. More specifically, one study found that midlife women that implemented regret-motivated life changes towards their life regret showed better well-being outcomes (Stewart and Vandewater, 1999). Additional research supports the adaptiveness of regret resolution as an effective coping mechanism, but further specifies that such engagement is adaptive primarily in circumstances where opportunities to undo the regret are favourable. For example, retirees who perceived few opportunities to resolve their regret and therefore disengaged exhibit better well-being (Farquhar et al., 2013). In turn, those who perceived high opportunity and therefore engaged in regret reversal experienced greater retirement satisfaction and increased daily activity levels, both at baseline and over time (Farquhar et al., 2013). Moreover, Wrosch et al. (2005) demonstrated that regret disengagement significantly predicted fewer regret-related intrusive thoughts, lower negative affect (i.e., lower regret intensity), fewer health problems, and fewer depressive symptoms.

#### Interpretation and appraisal

Research further suggests that changing one’s interpretation or appraisal of their life regret can have positive effects on well-being. For example, Dijkstra and Barelds (2008) found that women who fully accepted their life regret reported the highest psychological and physical well-being. Further, women who exhibited moderate acceptance reported intermediate well-being, followed by those who did not demonstrate any acceptance with the lowest physical well-being scores. Similarly, Torges et al. (2005) found that older adults who did not accept their regrets had the lowest levels of life satisfaction and positive affect, while, comparatively, those who “put the best face on things” towards their regrets reported better physical well-being. These studies highlight that attempting to accept life regrets can have significant impacts on psychological and physical well-being.

Other related lines of research suggest that altering one’s interpretation or appraisal of their life regret can also have significant positive effects on well-being. In addition, Wrosch et al. (2007) conducted an experimental study to assess the efficacy of a writing intervention in reducing regret and alleviating its adverse impact on the physical health of older adults. The study found that participants in the writing condition experienced reduced regret intensity over time, which in turn protected against the negative effect of regret intensity on sleep problems. Additionally, participants in the writing condition also experienced a decrease in regret-related hot emotions, (e.g., anger, irritation, and embarrassment) over time, suggesting that perhaps the mere act of reporting one’s life regret at baseline is sufficient to produce some therapeutic benefit.

#### Social comparison

During the process of life review, individuals might evaluate their successes by comparing it to the success of others. Indeed, the present review identified two studies which point to social comparison as a factor influencing both the severity of life regrets and their consequences on well-being. First, Seiden (2001) revealed that people with family or romantic relationship regrets who engaged in upward social comparisons tended to experience more severe regret and lower family satisfaction compared to those who engaged in downward social comparison. Similarly, Bauer and Wrosch (2011) found that for people who perceived low opportunities to overcome their regret, downward social comparisons led to increased positive affect and a reduction in cold symptoms over time.

#### Individual differences

The included literature further identifies several dispositional factors, including personality traits and cognitive patterns, that may underlie variations in the impact and experience of life regrets between individuals. Emotional stability is one such personality factor highlighted in the literature. Pethtel (2012) demonstrated a significant negative association between life regret intensity and levels of emotional stability. Similarly, studies have shown that neuroticism, an inverse measure of emotional stability, was significantly positively associated with life regret intensity and frequency (Reker and Woo, 2011; Isenberg, 2007). Moreover, emotional stability has been shown to mediate the relationship between age and emotional responses to life regret reflection (Tassone et al., 2019). As outlined by the literature, increased emotional stability with age is suggested to be an underlying factor in the association where older adults experience reduced regret-related negative affect when reflecting on their life regrets. Variations in cognitive patterns have also been associated with varying life regret experiences. For example, a study conducted by Schmidt and Van der Linden (2013) revealed that inducing regret reflection before sleep produced delays in sleep onset, but only for participants who habitually experienced high levels of counterfactual thoughts and emotions prior to sleep.

### Age-related findings

This section provides an overview of the age-related findings in the life regret and well-being literature. This section is broken up into three sections: regret content, trends, and coping.

#### Regret content

Several studies explore how the types of life regrets experienced vary across the lifespan. For example, it has been found that younger adults report more leisure and romantic regrets while older adults report more spirituality/religiosity and family-related regrets (Newton et al., 2012; Funderburk, 2008; Isenberg, 2007; Jokisaari, 2003, 2004; Lecci et al., 1994). However, when evaluating work-related regrets, the findings are mixed. Jokisaari (2003, 2004) found that middle-aged, and older adults reported more work-related regrets, while Isenberg (2007) found that younger adults have more work-related regrets. Additionally, Funderburk (2008) found that the oldest-old adults were less likely to have work and education regrets.

#### Trends

Several studies also explored age-related trends in the frequency, nature, and well-being consequences of life regrets. In this vein, Lecci et al. (1994) found that on average, people began to regret not having pursued past unfulfilled goals by 20 years old. Additionally, Reker and Woo (2011) found that reporting greater fear of aging was in fact associated with higher levels of existential regret. In contrast, several studies documented that across diverse older adult samples, life regret intensity decreases in old age (Isenberg, 2007; Kourakis, 2008; Neimeyer et al., 2011). Further, Wrosch and Heckhausen (2002) found that while the intensity of regret-related emotions did not vary with age, older adults did report less internal control over their regret. Consistently, Jokisaari (2003) also found that older adults evaluated their regrets as less likely to change, and less under their personal control. Further, internal control was positively associated with regret intensity and regret-related intrusive thoughts in older adults. Conversely, internal control was negatively associated with regret intensity and intrusive thoughts in younger adults. Finally, Newton et al. (2012) found that the more types of regrets middle-aged and older women report, the lower their life satisfaction. However, this pattern did not hold for younger women.

#### Coping

A handful of studies have also explored age-related patterns in coping with life regrets. Tassone et al. (2019) found that older adults reported relatively less negative emotion during life regret reflection compared to younger adults, an effect they suggest might be explained by increased emotional stability. Additionally, temporal distance from the life regret event was found to mediate the relationship between age and regret-induced negative emotions (Tassone et al., 2019). This finding suggests that older adults tend to experience more positive emotional responses during regret reflection in part due to a greater temporal distance from these events. In addition, Wrosch et al. (2005) found that older adults reported significantly fewer depressive symptoms and less intrusive thoughts (for commission regrets only), than younger adults. Further, disengagement from life regrets was found to protect older adults from increased depressive symptoms and health problems, effects that were mediated through decreased regret intensity (Wrosch et al., 2005). In this vein, Bauer and Wrosch (2011) found that the benefits of downward social comparisons in increasing positive affect were moderated by perceived opportunities to overcome the life regret, regardless of age.

### Gender/sex-related findings

In reporting gender and sex-related findings, we aimed to use the same wording as the original authors to the best of our ability. Importantly, most of the reviewed papers used terms interchangeably, making it challenging to differentiate between gender versus sex-based differences. Majority of the studies included in this review involved female dominant samples. Five studies (17%) included a fully female sample, and two (7%) involved a fully male sample. Overall, the literature suggests that women most frequently report life regrets related to educational pursuits, familial experiences, and romantic relationships (Dijkstra and Barelds, 2008; Jokisaari, 2004; Lecci et al., 1994; Metha et al., 1989; Newton et al., 2012; Stewart and Vandewater, 1999; Wrosch and Heckhausen, 2002). Moreover, the research indicates that men most frequently report life regrets regarding education and work (Jokisaari, 2004; Lecci et al., 1994; Wrosch and Heckhausen, 2002). While most studies show no gender differences in the number, frequency, or intensity of reported life regrets (Isenberg, 2007; Lecci et al., 1994; Newall et al., 2009; Seiden, 2001; Torges et al., 2005), some demonstrated significant gender differences in various life regret dimensions. For example, two studies found that men are more likely than women to report having a life regret experience (Funderburk, 2008; Neimeyer et al., 2011). Further, Choi and Jun (2009) found that women experience more intense family-related regrets, and Seiden (2001) showed that women with family and romantic regrets suffer greater declines in personal life quality and family life satisfaction compared to men with similar regrets. Some evidence shows that women report having less control (Isenberg, 2007), and fewer opportunities to reverse their life regrets (Farquhar et al., 2013). Other studies report no such gender differences in ratings of controllability, changeability or consequence (Choi and Jun, 2009; Jokisaari, 2003; Torges et al., 2005).

A few studies focused exclusively on female participants, aiming to examine various dimensions of female-specific regret experiences. For example, Stewart and Vandewater (1999) found that females with life regrets tied to traditional gender roles who successfully made regret-related changes experienced improved well-being outcomes, despite facing similar contextual barriers (i.e., family responsibilities) as those who did not make such changes. Lewis and Borders (1995) investigated a fully female-identifying sample exploring the role of gender identity in life regret experiences beyond a gender binary, examining sex role orientation dimensionally. A negative association between increased masculine traits and life regret emerged, although sex role orientation was not significantly related to life satisfaction. In contrast, research focused on fully male samples investigated rather unique samples and different well-being variables. Kourakis (2008) discovered that increased life regret was significantly associated with higher levels of depression, worse physical health, and more death anxiety in older male veterans. Further, Seiden (2001) found that middle-aged married men with children who reported romantic relationships and personal regrets had the lowest quality of personal life.

## Discussion

The present systematic review identified, summarized, and analyzed the 31 empirical articles examining the association between life regrets and well-being. Across these studies, life regret was consistently linked to diminished well-being. This negative association was demonstrated across various dimensions of regret (e.g., the presence of life regret, the number of life regrets, the intensity of these regrets, and their frequency) and a diverse array of psychological, emotional, and physical well-being indicators. Further, the literature highlights that the impact of life regret on well-being depends on the way in which people interpret and manage their life regrets. In the following section, we will discuss and critically assess this literature across four themes, pointing to gaps and suggestions for future directions.

### Sample characteristics

Over the past few decades of psychological research, there has been significant criticism of the predominance of Western, Educated, Industrialized, Rich and Democratic (WEIRD) samples (Henrich, 2020; Henrich et al., 2010). Heavily relying on WEIRD participants to develop empirically informed assumptions and psychological explanations of human behaviour becomes limited when lacking diverse samples (de Oliveira and Baggs, 2023). To the best of our knowledge, most studies included in this review primarily focused on white, highly educated populations, thus limiting the generalizability of the present findings to non-WEIRD individuals. However, over half of the reviewed studies neglected to report the participant ethnicities in their sample, making it unclear to which population the results are generalizable. Of note, Choi and Jun (2009) focused specifically on life regret in a low-income, ethnically diverse sample, and found that demographics like socioeconomic status may be indicative of what domain people tend to regret the most. Further, Lee and Ryu (2018) examined cultural differences directly and found differential experiences and impacts of regret between Americans and Koreans. Together these studies highlight the need to prioritize future research in diverse samples.

On the other hand, while samples in psychological research typically skew very young, that is not the case in the presently reviewed literature, with several studies focused on life regret across the lifespan, recruiting participants in diverse age groups. Therefore, we consider it a strength of the review literature, as the inclusion of full age ranges has provided a more comprehensive understanding of life regret throughout adulthood. That being said, age-related changes in life regret were mixed, with much variability observed across different regret domains and perceived levels of internal control. However, across three studies, there was an overlap between regret intensity and aging, such that regret intensity tended to decrease with age (Isenberg, 2007; Kourakis, 2008; Neimeyer et al., 2011). Regret is often associated with (and even operationalized as) negative emotions. Research indicates older adults tend to experience reduced negative affect (Kessler and Staudinger, 2009) and are less expressive of negative emotions compared to younger adults (Phillips et al., 2006). Thus, age-related differences in emotional processes may inform the lower regret intensities observed in older adults. To clarify the mixed age-related findings, future research should explore how older adults manage negative emotions brought by their regret and how the depth of lived experience influences life regret experiences.

Furthermore, mixed findings between life regrets and gender emerged. Several studies found the regret domain to be of notable variation. Aside from education, which was consistently cited by both genders, women commonly reported family-related life regrets, while men more frequently reported work-related regrets. However, as 26 of the reviewed studies were published over a decade ago, the limited recency of research and scarcity of replication may perpetuate gender-specific trends that are no longer accurate in current contexts. Like other dimensions of human experience, the nature of life regrets has likely shifted, reflecting changes in societal values—especially relative to gender expectations and stereotypes. Newton et al. (2012) illustrated this concept by demonstrating that older cohorts of women often have traditional life regrets related to family, whereas women born after the 1960s Women’s Movement tended to have more life regrets related to education and career. Gender-specific trends in the experience of life regret (i.e., number, frequency, intensity) remain mixed and vary between each study, with limited to no overlap. Inconsistent measures and excessive variance in the results make it challenging to understand how life regret manifests differently across genders, highlighting the need for updated research.

### Measurement of regret

Importantly, there are considerable variabilities in the operationalization of this construct across the literature. Some studies focus solely on the presence or absence of life regret, while others examine more specific dimensions (e.g., content, number, intensity). Further, even among studies examining the same dimensions of regret, there are notable differences in the measurement of these constructs.

Despite using common labels to describe similar variables, the differing scales employed in each study may have led to the measurement of different aspects of those variables, making comparisons across the studies challenging. This reflects the Jingle-Jangle Fallacy, described by Marsch (1994), which refers to the phenomenon where scales measuring distinct constructs use the same label. These variations make it difficult to directly compare findings across studies and draw meaningful, generalized conclusions about specific aspects of life regret experiences. In this vein, this disorganized diversity of methods and variability in the measurement of life regret across the literature hampers the ability to generalize findings and impacts the reliability and validity of research in this field, highlighting the need for the development of a more standardized approach.

### Well-being

Subjective well-being is a multi-dimensional concept that is often assessed and interpreted using a multitude of methods (Jarden and Roache, 2023). A myriad of well-being measures were used to capture levels of well-being and overall quality of life throughout the literature. Despite considerable variability in the well-being indicators used across the reviewed studies, nearly all scales were negatively associated with life regret. Life satisfaction was the most common factor used to reflect well-being, as major regrets often indicate areas where individuals believe an alternative outcome would have led to greater satisfaction with their current life (e.g., Torges et al., 2005). Measures of mental health (e.g., depression and anxiety) and emotional well-being (e.g., positive and negative affect) similarly followed this negative trend. Notably, none of the reported research examined the association between life regrets and the components of Ryff’s (1989) conception of psychological well-being. Future research would benefit from such analyses to further clarify the potentially differential impact of life regrets across these components.

Physical health status was also a common measure of well-being, particularly in aging samples. Again, there was much variability in the way that physical health was measured (e.g., general health status, quality of sleep), making it challenging to draw clear conclusions due to the wide range of findings. In this vein, the interconnection between psychological and physical well-being may underlie the impact of life regrets on physical health (Cho et al., 2011). While substantial evidence suggests a bi-directional association, where higher levels of psychological well-being can strengthen physical health through motivation and where being in good physical health also promotes a healthy lifestyle that enhances psychological well-being (Granero-Jiménez et al., 2022), there is currently not enough evidence to confirm this hypothesis.

Finally, given the few studies using longitudinal (*n* = 3) or experimental (*n* = 2) designs, there is not sufficient evidence to support directionality in the relationship between life regrets and well-being. It is unclear whether life regrets have adverse effects on well-being or whether experiencing worse well-being leads individuals to more frequently look back on their lives and reflect on the things they regret.

### Protective factors

The reviewed literature highlights the importance of adaptive behavioural and psychological coping mechanisms in protecting overall well-being. Of note, the existing literature highlights the complementary roles of both engagement and disengagement. For example, some results point to well-being improvements associated with engaging in efforts to undo the life regret (Stewart and Vandewater, 1999), while others focus on the beneficial effects of disengaging psychologically from the regret (Wrosch et al., 2005). These effects were often shown to be contingent on perceived opportunities (i.e., the likelihood that regret can and will be undone) to undo the regret. When opportunities were high, engagement in undoing the regret was adaptive. Alternatively, when opportunities were low, disengaging from the regret through acceptance or downward social comparison proved to be adaptive. In this way, it is suggested that it may not be age, or even actual opportunities to overcome their regrets that impacts well-being, but rather the perception of opportunities.

Variations in coping with major regret may inform the gap between *why* life regret is repeatedly linked to adverse well-being. For example, research on goal disengagement suggests that disengaging from an unattainable goal that holds personal value can alleviate feelings of helplessness, ultimately increasing quality of life (Koppe and Rothermund, 2017; Wrosch et al., 2003). These findings may hold true to life regret, as it is often related to a significant personal goal in a valued life domain. However, the mechanisms through which experiencing life regret produces negative effects on well-being are not clear from this scope of literature. Further, it remains unclear as to which strategies are most effective in managing life regret, and potential mediating factors in this association remain relatively unexplored. Consequently, future research must explore the well-being implications of attempting to repair a life regret versus disengaging when reversal is not possible. Identifying this distinction and examining its long-term effects on well-being may allow researchers to uncover effective interventions and strategies for coping with and reappraising life regrets.

## Conclusion

Overall, the present systematic review points to a culmination of research suggesting that experiencing life regret is negatively related to well-being. A meta-analysis of the present scope of literature is not currently feasible due to a lack of homogeneity across regret measures, thus, we chose to perform a systematic review to describe the available research on the relationship between life regret and well-being. The development of a more standardized approach to measuring life regrets is needed in future research to enhance the coherence and utility of research on life regret. However, despite this variability in the measurement of regret, the uniformity of findings across studies underscores the reliability and strength of the overall conclusions made in the present review. This review has implications for theories of well-being and aging, as well as the potential to inform intervention development.

## Data Availability

The datasets presented in this study can be found in online repositories. The names of the repository/repositories and accession number(s) can be found in the article/supplementary material.

## Footnotes

^1^Importantly, the present review focuses on regret specifically in the context of the lifespan, as a life reflection process. The concept of regret is examined in various other contexts throughout existing literature. For example, experimental studies involving tasks designed to elicit regret (e.g., gambling tasks) were not deemed relevant to the present conceptualization, as situational regret is unlikely to elicit persistent, long-term regret relevant in the context of the lifespan. In addition, many studies investigated regret experiences regarding healthcare-related decisions and outcomes. While these experiences of regret might be considered severe and pervasive in the context of the lifespan, regret experiences were not assumed to be life regrets unless explicitly framed as such. As such, the present review conceptualizes life regret as a broad range of people’s recalled regret towards major life events, such as career choices, relationships, family, or personal behaviour (Choi and Jun, 2009; Jokisaari, 2004; Gilovich and Medvec, 1995; Wrosch and Heckhausen, 2002).

^2^While language was not included as an inclusion criterion in the original pre-registration, the authors came to realize the necessity in the screening process. Importantly, however, to our knowledge, this only excluded one study (Junaković et al., 2018).
